# Supplementary material for: AlphaFold3-based modeling uncovers the dynamic structural interface between full-length IAP antagonists and DIAP1 for apoptosis regulation in Drosophila
Source: bioRxiv. 2026 Jan 10:2026.01.09.698746. Preprint. [Version 1] doi: 10.64898/2026.01.09.698746 (PMC12803136; doi:10.64898/2026.01.09.698746)

Suppl Fig S1

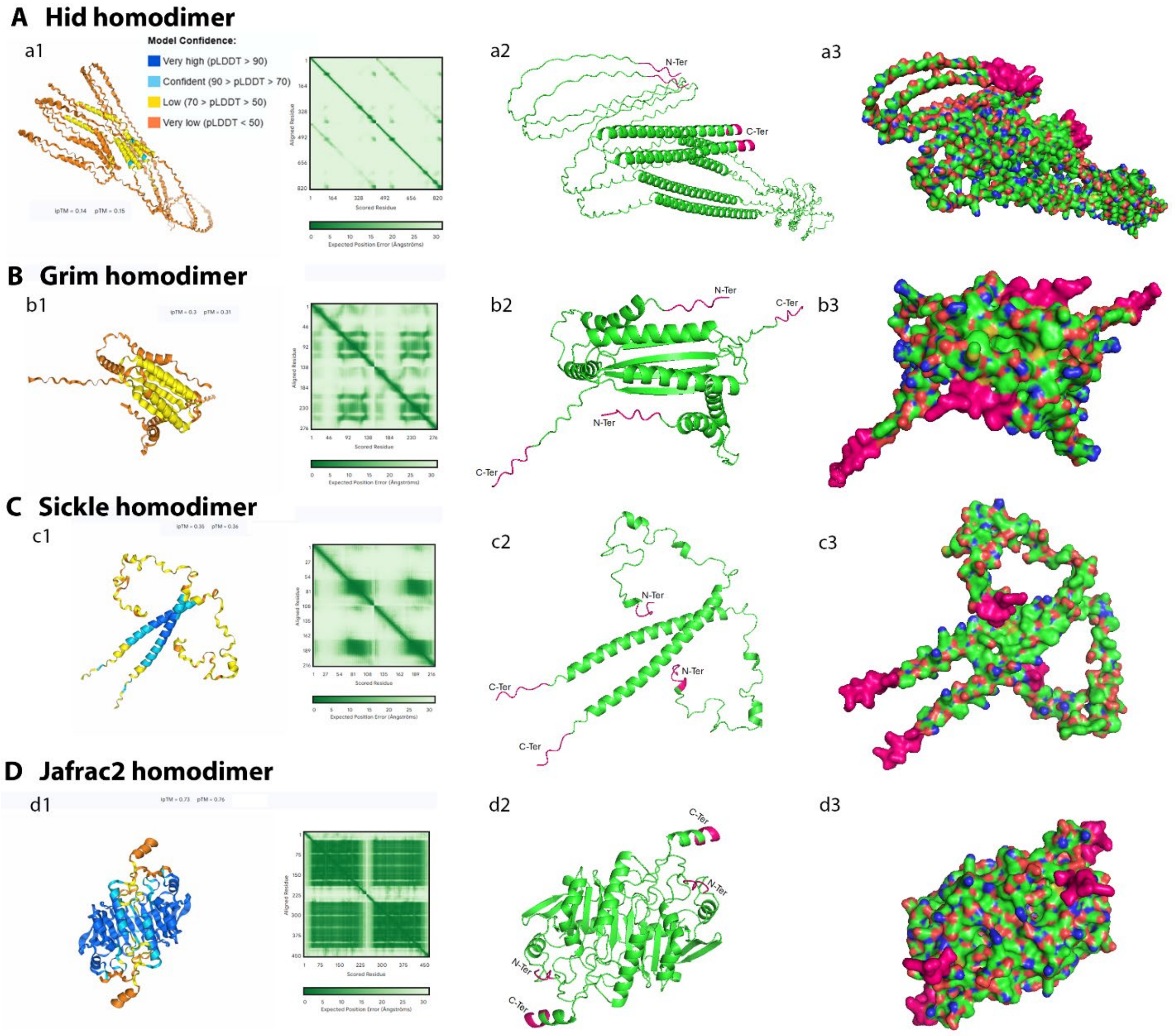
A Hid homodimer
a1
Model Confidence:
Very high (pLDDT > 90)
Confident (90 > pLDDT > 70)
Low (70 > pLDDT > 50)
Very low (pLDDT < 50)
ipTM = 0.14 pTM = 0.15
Aligned Residue
Scored Residue
Expected Position Error (Ångströms)
a2
N-Ter
C-Ter
a3
B Grim homodimer
b1
b2
N-Ter
C-Ter
b3
C Sickle homodimer
c1
c2
N-Ter
C-Ter
c3
D Jafrac2 homodimer
d1
d2
C-Ter
N-Ter
d3

Suppl Fig S2

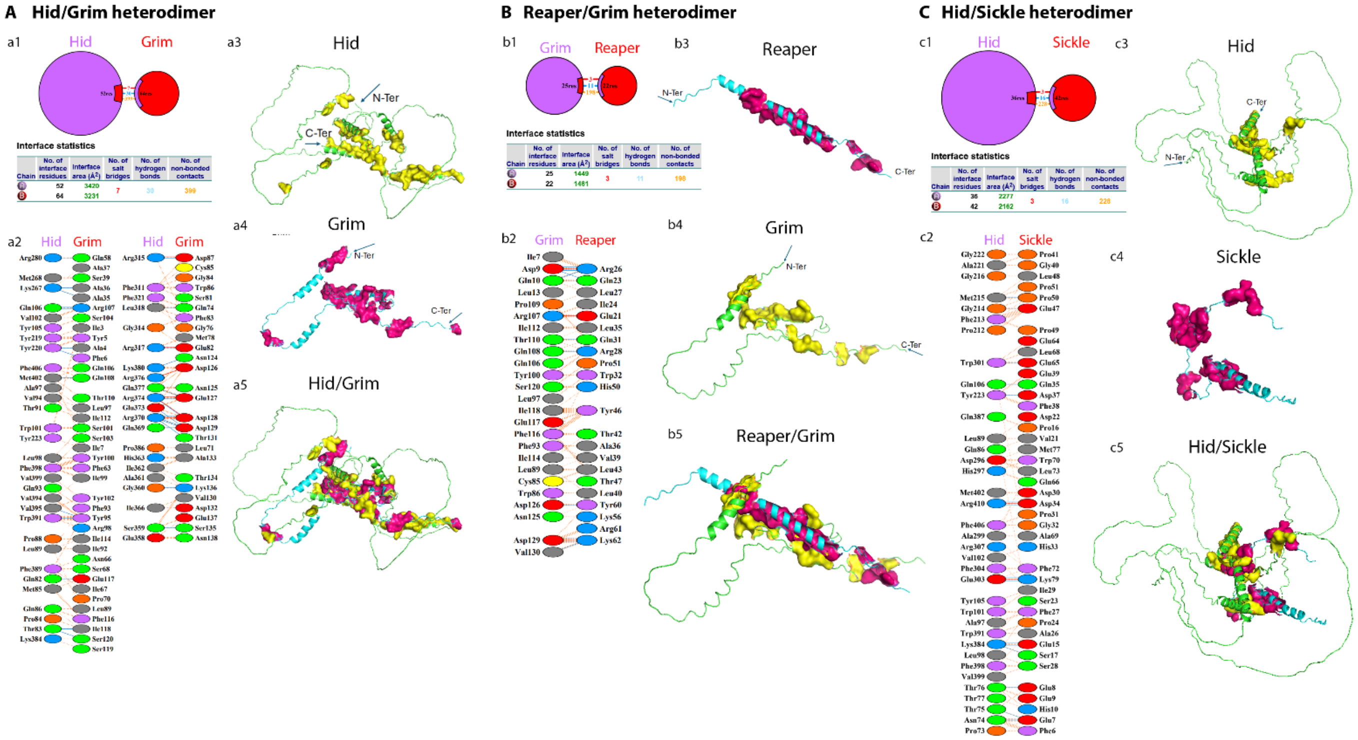
A Hid/Grim heterodimer
a1
Hid
Grim
Interface statistics
a2
a3
Hid
N-Ter
C-Ter
a4
Grim
a5
Hid/Grim
B Reaper/Grim heterodimer
b1
Grim
Reaper
Interface statistics
b2
b3
Reaper
b4
Grim
b5
Reaper/Grim
C Hid/Sickle heterodimer
c1
Hid
Sickle
Interface statistics
c2
c3
Hid
c4
Sickle
c5
Hid/Sickle

Suppl Fig S3

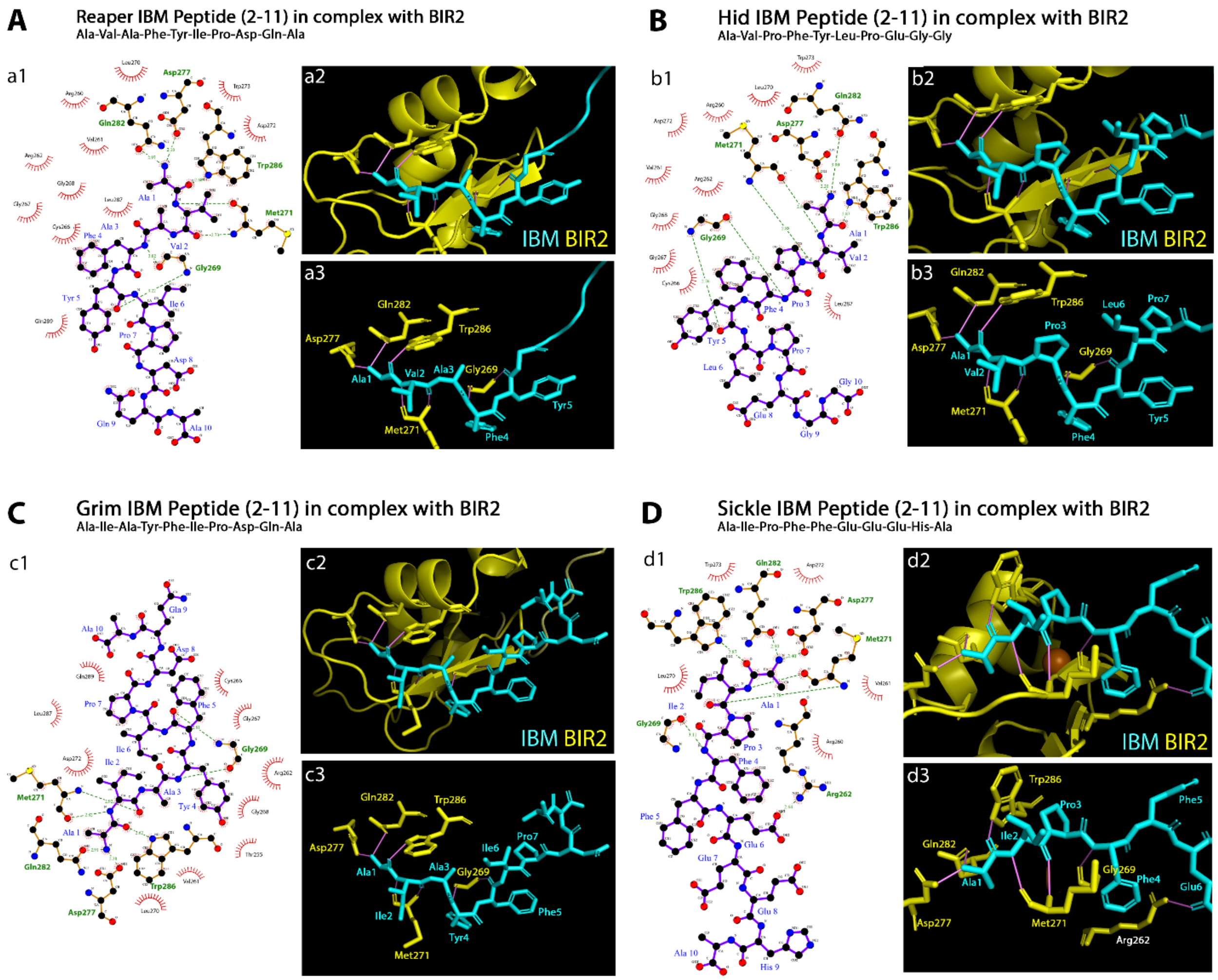

A
Reaper IBM Peptide (2-11) in complex with BIR2
Ala-Val-Ala-Phe-Tyr-Ile-Pro-Asp-Gln-Ala
a1
a2
IBM BIR2
a3
Gln282
Trp286
Asp277
Ala1
Val2
Ala3
Gly269
Tyr5
Met271
Phe4
B
Hid IBM Peptide (2-11) in complex with BIR2
Ala-Val-Pro-Phe-Tyr-Leu-Pro-Glu-Gly-Gly
b1
b2
IBM BIR2
b3
Gln282
Trp286
Leu6
Pro7
Pro3
Asp277
Ala1
Gly269
Val2
Met271
Tyr5
Phe4
C
Grim IBM Peptide (2-11) in complex with BIR2
Ala-Ile-Ala-Tyr-Phe-Ile-Pro-Asp-Gln-Ala
c1
c2
IBM BIR2
c3
Gln282
Trp286
Asp277
Ile6
Pro7
Ala1
Ala3
Gly269
Ile2
Phe5
Tyr4
Met271
D
Sickle IBM Peptide (2-11) in complex with BIR2
Ala-Ile-Pro-Phe-Phe-Glu-Glu-Glu-His-Ala
d1
d2
IBM BIR2
d3
Trp286
Phe5
Pro3
Gln282
Ile2
Ala1
Gly269
Phe4
Glu6
Asp277
Met271
Arg262

Suppl Fig S4

**A** DIAP1/IBM Reaper (2-11aa)

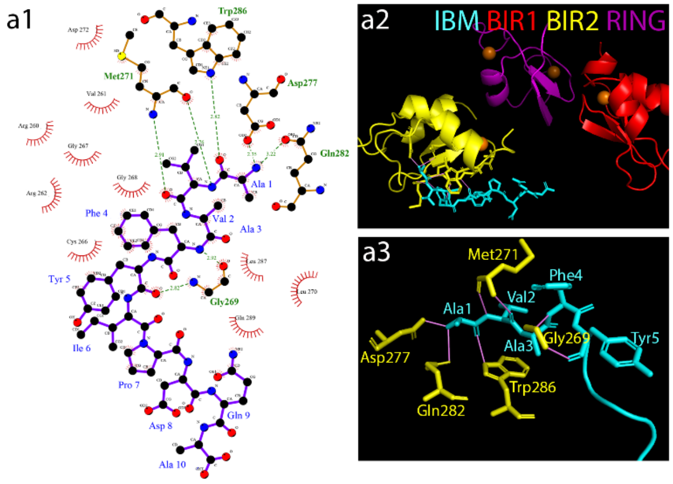


**B** DIAP1/IBM Grim (2-11 aa)

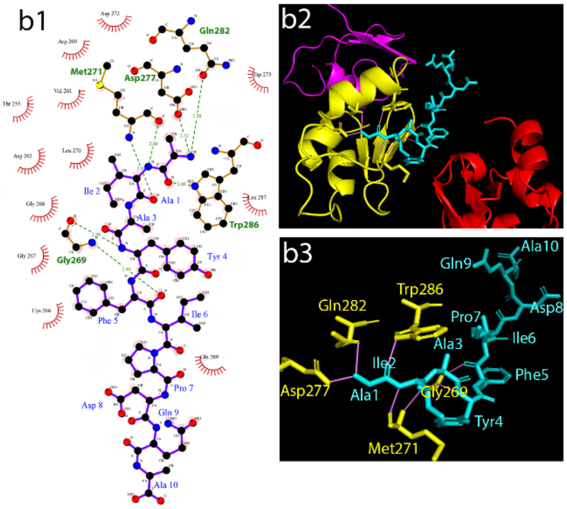


**C** DIAP1/IBM Hid (2-11 aa)

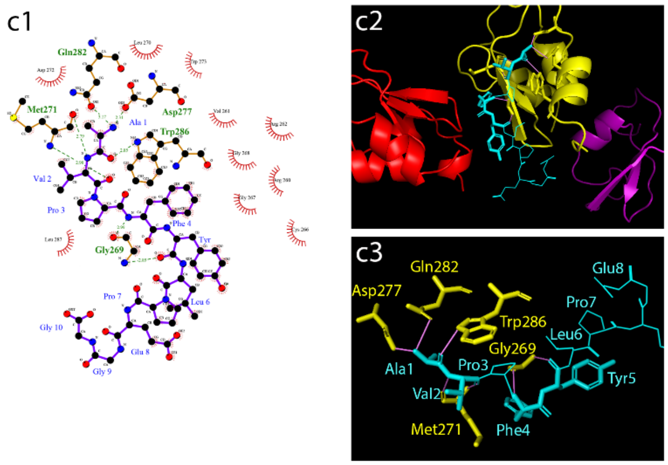


**D** DIAP1/IBM Sickle (2-11 aa)

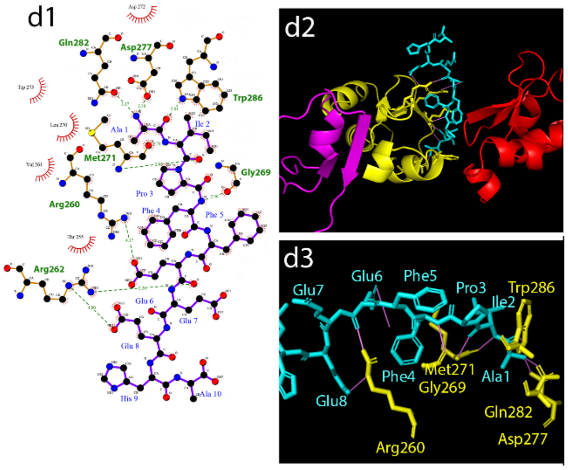

Suppl Fig S5

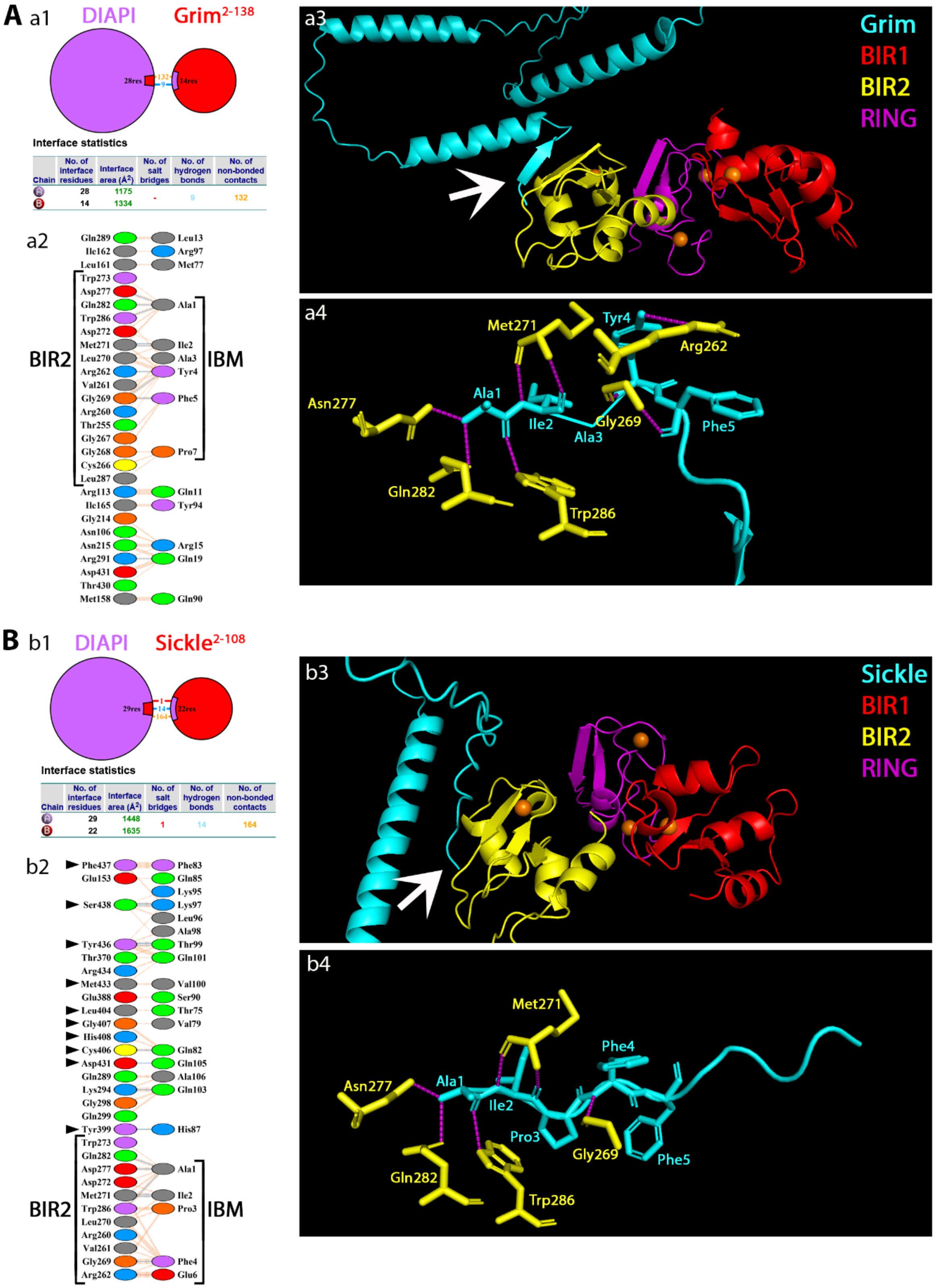
A a1
DIAPI
Grim2-138
28res
14res
132
9
Interface statistics
Chain | No. of interface residues | Interface area (Å2) | No. of salt bridges | No. of hydrogen bonds | No. of non-bonded contacts
A | 28 | 1175 | - | 9 | 132
B | 14 | 1334
a2
Gln289 Ile162 Leu161 Trp273 Asp277 Gln282 Trp286 Asp272 Met271 Leu270 Arg262 Val261 Gly269 Arg260 Thr255 Gly267 Gly268 Cys266 Leu287 Arg113 Ile165 Gly214 Asn106 Asn215 Arg291 Asp431 Thr430 Met158
Leu13 Arg97 Met77 Ala1 Ile2 Ala3 Tyr4 Phe5 Pro7 Gln11 Tyr94 Arg15 Gln19 Gln90
BIR2
IBM
a3
Grim
BIR1
BIR2
RING
a4
Met271
Tyr4
Arg262
Asn277
Ala1
Ile2
Gly269
Ala3
Phe5
Gln282
Trp286
B b1
DIAPI
Sickle2-108
29res
22res
1
14
164
Interface statistics
Chain | No. of interface residues | Interface area (Å2) | No. of salt bridges | No. of hydrogen bonds | No. of non-bonded contacts
A | 29 | 1448 | 1 | 14 | 164
B | 22 | 1635
b2
Phe437 Glu153 Ser438 Tyr436 Thr370 Arg434 Met433 Glu388 Leu404 Gly407 His408 Cys406 Asp431 Gln289 Lys294 Gly298 Gln299 Tyr399 Trp273 Gln282 Asp277 Asp272 Met271 Trp286 Leu270 Arg260 Val261 Gly269 Arg262
Phe83 Gln85 Lys95 Lys97 Leu96 Ala98 Thr99 Gln101 Val100 Ser90 Thr75 Val79 Gln82 Gln105 Ala106 Gln103 His87 Ala1 Ile2 Pro3 Phe4 Glu6
BIR2
IBM
b3
Sickle
BIR1
BIR2
RING
b4
Met271
Phe4
Asn277
Ala1
Ile2
Pro3
Gly269
Phe5
Gln282
Trp286

Suppl Fig S6

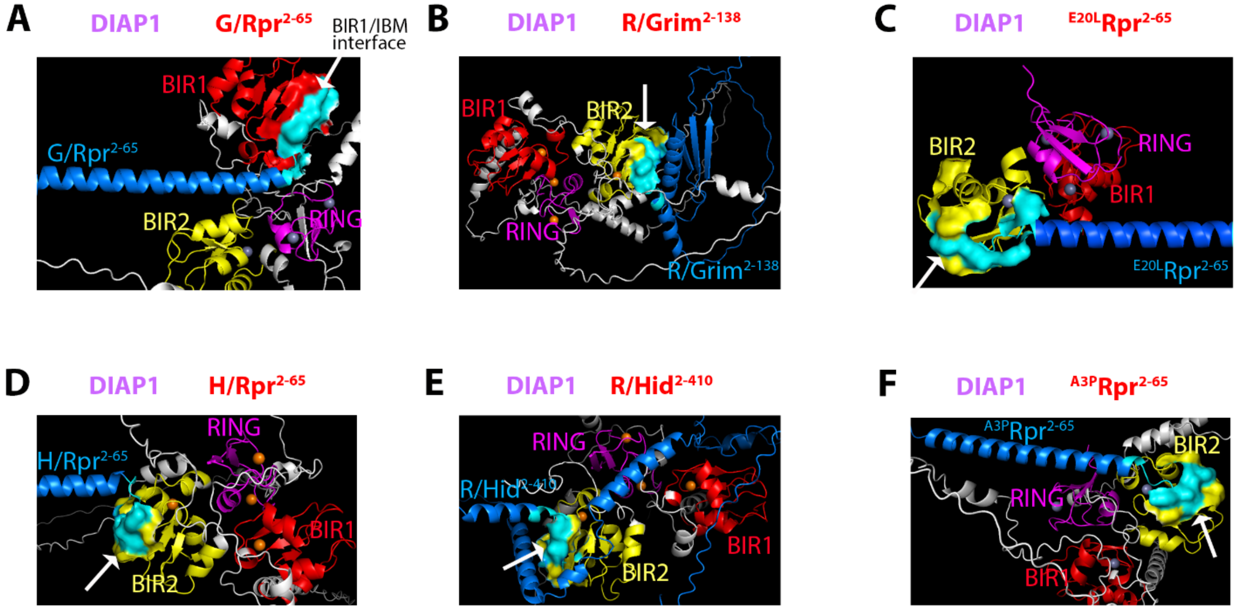
A
DIAP1
G/Rpr2-65
BIR1/IBM interface
BIR1
G/Rpr2-65
BIR2
RING
B
DIAP1
R/Grim2-138
BIR1
BIR2
RING
R/Grim2-138
C
DIAP1
E20LRpr2-65
BIR2
RING
BIR1
E20LRpr2-65
D
DIAP1
H/Rpr2-65
RING
H/Rpr2-65
BIR1
BIR2
E
DIAP1
R/Hid2-410
RING
BIR1
BIR2
F
DIAP1
A3PRpr2-65
BIR2
RING

Suppl Fig S7

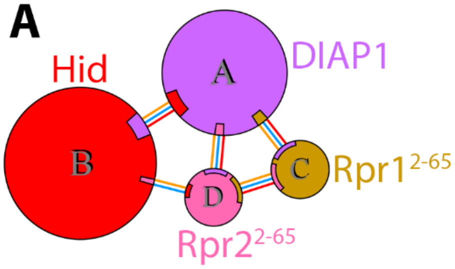


**Interface statistics**

| Chains | No. of interface residues | Interface area ($Å^2$) | No. of salt bridges | No. of hydrogen bonds | No. of non-bonded contacts |
|---|---|---|---|---|---|
| A–B | 52:35 | 2423:2757 | 4 | 16 | 365 |
| A–C | 16:17 | 1044:1165 | 3 | 8 | 171 |
| A–D | 21:14 | 796:1017 | 1 | 8 | 163 |
| B–D | 11:11 | 595:589 | - | 2 | 143 |
| C–D | 21:21 | 1234:1191 | 1 | 6 | 347 |

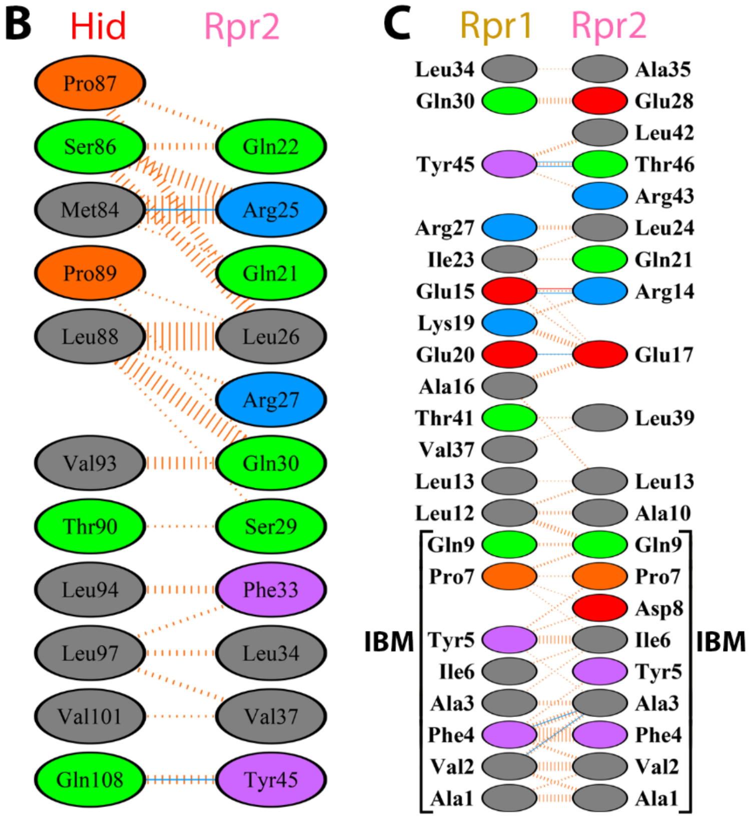

Supplement: Supplement 1 — Supplementary Figure S1. Dimer models of Hid, Grim, Sickle, and Jafrac2. (related to Figure 2) AF3-predicted models of the dimers of Hid (A), Grim (B), Sickle (C) and Jafrac2 (D). Confidence values vary across interfaces and are low except for the Jafrac2 dimer. Supplementary Figure S2. Heterodimeric interactions between IAP antagonists. (related to Figure 2) AF3-predicted models of (A) Hid/Grim, (B) Rpr/Grim, and (C) Hid/Sickle heterodimeric complexes. The a1, a2, b1, b2, c1 and c2 panels illustrate the protein-protein interfaces, interface statistics and residue interactions at the interfaces of the heterodimers according to PDBsum. The a3-a5, b3-b5 and c3-c5 panels show PyMOL-generated structures of the heterdimeric complexes. Hid and Grim (b4) are colored in green with the interacting residues highlighted in yellow. Rpr, Grim (a4) and Sickle are shown in cyan with the interacting residues highlighted in red. Supplementary Figure S3. IBM peptide interactions with isolated BIR2. (related to Figure 4) (A-D) PDBsum- and PyMOL-generated images of the interaction of the IBM peptides (10mers) of Rpr (A), Hid (B), Grim (C) and Sickle (D) with the isolated BIR2 domain (residues 201 to 325) of DIAP1. (a1, b1, c1, d1) The PDBsum-generated diagrams present a 2D interaction map with the IBM peptide shown in the center. IBM residues are labeled in blue, while residues from the BIR2 domain that engage in hydrogen bonding with the peptide are labeled in green. Hydrogen bonds are indicated as green dashed lines. Hydrophobic contacts are depicted as spoked arcs (half-circles with radiating lines), drawn around BIR2 residues whose side chains form non-bonded interactions with IBM residues. A complete list of all hydrogen bonds and non-bonded contacts is provided in Supplementary Table S1. (a2, a3, b2, b3, c2, c3, d2, d3) PyMOL-rendered images depicting the interaction between the IBM peptides and the BIR2 domain. The BIR2 domain is shown in yellow, the IBM peptide in cyan. Hydroge [file media-1.pdf]
